# A Deep-Learning-Based Approach for Wheat Yellow Rust Disease Recognition from Unmanned Aerial Vehicle Images

**DOI:** 10.3390/s21196540

**Published:** 2021-09-30

**Authors:** Qian Pan, Maofang Gao, Pingbo Wu, Jingwen Yan, Shilei Li

**Affiliations:** 1 Key Laboratory of Digital Signal and Image Processing of Guangdong Province, Shantou University, Shantou 515063, China; 19qpan@stu.edu.cn (Q.P.); 19pbwu@stu.edu.cn (P.W.); jwyan@stu.edu.cn (J.Y.); 2 Key Laboratory of Agricultural Remote Sensing, Ministry of Agriculture and Rural Affairs/Institute of Agricultural Resources and Regional Planning, Chinese Academy of Agricultural Sciences, Beijing 100081, China; 82101201641@caas.cn

**Keywords:** generalization ability, PSPNet, UAV image, weak label, weakly supervised learning, wheat yellow rust

## Abstract

Yellow rust is a disease with a wide range that causes great damage to wheat. The traditional method of manually identifying wheat yellow rust is very inefficient. To improve this situation, this study proposed a deep-learning-based method for identifying wheat yellow rust from unmanned aerial vehicle (UAV) images. The method was based on the pyramid scene parsing network (PSPNet) semantic segmentation model to classify healthy wheat, yellow rust wheat, and bare soil in small-scale UAV images, and to investigate the spatial generalization of the model. In addition, it was proposed to use the high-accuracy classification results of traditional algorithms as weak samples for wheat yellow rust identification. The recognition accuracy of the PSPNet model in this study reached 98%. On this basis, this study used the trained semantic segmentation model to recognize another wheat field. The results showed that the method had certain generalization ability, and its accuracy reached 98%. In addition, the high-accuracy classification result of a support vector machine was used as a weak label by weak supervision, which better solved the labeling problem of large-size images, and the final recognition accuracy reached 94%. Therefore, the present study method facilitated timely control measures to reduce economic losses.

## 1. Introduction

Wheat yellow rust is characterized by wide distribution, rapid spread, and a large damage area in wheat production in China. The traditional field monitoring approach for crop diseases is labor-intensive, subjective, and less efficient. With the rapid development of remote-sensing (RS) technology, aerial remote sensing is gradually becoming an important complementary approach. This approach of using an unmanned aerial vehicle (UAV) to obtain RS data for relevant research is also gradually becoming widely used for crop disease identification, and makes it possible to identify crop diseases at a coarse scale.

Many scholars have conducted research related to how to achieve efficient identification of crop diseases from several perspectives and using traditional methods. Initially, many experts established inverse models of wheat yellow rust using spectral reflectance values collected with an Analytical Spectra Devices (ASD) spectrometer, and processed by various methods such as spectral derivative transform, continuous removal transform, and continuous wavelet transform. However, different processing methods and different inversion algorithms had a large impact on the models [1,2,3,4]. Later, Zhang et al. [5] used GF-1/WFV images to estimate wheat yellow rust conditions using the winter wheat stripe rust index (WSRI). The method was simple and operable, but the overall accuracy (OA) was not high and, roughly, the minimum value of the green light band and the maximum value of the near-infrared (NIR) band of winter wheat image elements at different normalized difference vegetation index (NDVI) growth levels were used as the reflectance values of the green and NIR bands of healthy winter wheat, without fully following the WSRI formula. Su et al. [6] used a random forest (RF) algorithm to identify healthy wheat and yellow rust wheat on UAV images of 1 to 1.5 cm/pixel. This study obtained yellow rust wheat of different severities by autonomous inoculation with yellow rust disease. Zhang et al. [7] established a method to quantitatively characterize the shape features around specific absorption locations in the spectrum by artificially inoculating wheat with powdery mildew fungus after nutrient stress treatment, based on the differences in canopy spectral reflectance of wheat with different disease conditions. The method was effective for the detection of wheat powdery mildew. Bohnenkamp et al. [8] artificially inoculated wheat with different concentrations of wheat yellow rust bacteria for wheat yellow rust identification using spectral angle mapper and support vector machine (SVM) methods at both canopy and UAV scales. The proximity of the areas of their multiple control experiments in these studies that relied on artificial inoculation with yellow rust bacteria resulted in inaccuracy of the collected severity data due to the spread of spores of wheat yellow rust that was not taken into account. Zhao et al. [9] used discriminant partial least squares, BPNN, and SVM for identification. In their study, the number of NIR spectra of several crop diseases varied widely, causing the identification accuracy to also vary widely, so the effectiveness of the discriminant partial least squares and SVM algorithms in the identification task could not be fully determined. In the current situation, traditional model inversion algorithms are not effective enough to meet the current needs of identifying crop diseases, and the emergence of methods that can efficiently identify crop diseases is required.

In response to the problems arising from traditional methods, some scholars have conducted corresponding research using deep-learning methods. Driven by big data, deep learning has been widely used in many fields such as computer vision and speech recognition [10]. The neural network model, which is one of the most representative models [11], is gradually being applied to the image classification study of aerial remote sensing. Gong et al. [12] proposed a D-CNN model that was trained by optimizing a new discriminative objective function and adding a metric learning regularization term to the CNN features, and this approach effectively improved the classification performance of remote sensing of image scenes. Gong et al. [13] proposed a novel feature-enhancement network for object detection in remote-sensing images. Context encoding loss was also used to normalize model training to facilitate better understanding of the scene by the object detector. Yao et al. [14] gradually selected simple samples from an unlabeled image dataset, assigned pseudo-labels to them, and further employed the labelled images as a new training set from which to learn, which increased the confidence level of the selected samples and facilitated the training of a more robust classifier. These studies have been conducted on aerial remote-sensing datasets with the aim of detecting objects such as aircraft and ships in the images. There is also a significant amount of research for the agricultural sector. Leng et al. [15] first proposed the use of UAVs to monitor wheat yellow rust and explored the feasibility of this technology. Zhang et al. [16] used a deep convolutional neural network model for wheat yellow rust identification on high-resolution hyperspectral images, and the study achieved high performance only in the late growth stage of wheat. In addition, labeling the categories with the magnitude of the average vegetation index of each block after cutting caused large errors; labeling the categories based on the pixels of the images would be more appropriate. Ocer et al. [17] used mask R-CNN and feature pyramid network models to extract trees from high-resolution drone data. This combination of shallow semantic value information with deep semantic value information improved the performance when identifying trees from UAV images. Su et al. [18] proposed a framework for monitoring wheat yellow rust by combining a U-Net semantic segmentation model and UAV multispectral and RGB images. However, the study was not accurate enough to achieve a large-scale replication due to the scarcity of labeled data. Liu et al. [19] established a monitoring model for wheat blast based on an improved BPNN. Comparison with RF, SVM, and partial least squares regression showed the stability and high identification accuracy of the model. However, the number of plots in this study was small, and the model validation process based on real independent data was lacking. Duarte-Carvajalino et al. [20] trained a neural network with a dataset extracted from image data of potatoes with late blight captured by UAV to identify the severity of the late blight. The study did not clearly distinguish other stressors that might affect the spectral characteristics of potatoes. Ha et al. [21] proposed a convolutional neural network (CNN) model for identifying *Fusarium*-wilt-infected radishes captured by a high-resolution UAV RGB camera, identifying healthy radishes and *Fusarium*-wilt-infected radishes with 93% accuracy. The study relied entirely on manual labeling of a large number of samples for training, which was very time consuming. Huang et al. [22] used UAV images to identify the severity of Helminthosporium leaf blotch disease of wheat, trained using CNN, and obtained an accuracy of up to 94%. In addition, they compared the performance of CNN with traditional methods and found that the CNN models performed better in identifying and classifying disease infestations. The study had some risk of overfitting and did not address this issue. Feng et al. [23] used weakly supervised learning to classify remote-sensing images and proposed a novel end-to-end progressive contextual instance refinement method that effectively improved the detection accuracy.

There were some problems in the above study. First, leaf-scale studies are not applicable to yellow rust identification in field wheat, and studies with artificial inoculation of yellow rust bacteria suffer from poorly controlled trial setups. Second, the low resolution and long revisit period of satellite images are not enough to provide real-time feedback on crop diseases in smaller plots, while aerial remote-sensing data such as that from UAVs can achieve fast, accurate, and lossless identification. In addition, some studies showed more misclassification between healthy and diseased crops, and could not achieve effective identification among neighboring pixels of healthy and diseased crops. Finally, in the case of a large-scale farm image or a large amount of image data, it is time consuming to rely entirely on manual labeling of samples. In addition, most of the studies did not illustrate or validate the generalizability of the model with experiments.

In this study, we used the PSPNet semantic segmentation model to identify healthy wheat, yellow-rust-affected wheat, and bare soil areas in a wheat field in Xigolou Village, Yuzhou City, Henan Province, and investigated the spatial generalization ability of this model in a wheat field in Dahuai Village of the same city. In addition, we proposed a weakly supervised learning approach to effectively solve the sample labeling problem and validated this approach. The rest of the paper is organized as follows: Section 2 introduces the study area and data sources; Section 3 details the methodology; Section 4 outlines the results and analysis; and Section 5 and Section 6 contain the discussion and conclusion, respectively.

## 2. Study Area and Data

This section describes the materials related to this study, including the location of the experimental area, the acquisition of high-resolution UAV RGB images, and the use of the image preprocessing software PhotoScan.

### 2.1. Study Area

**Figure 1:**
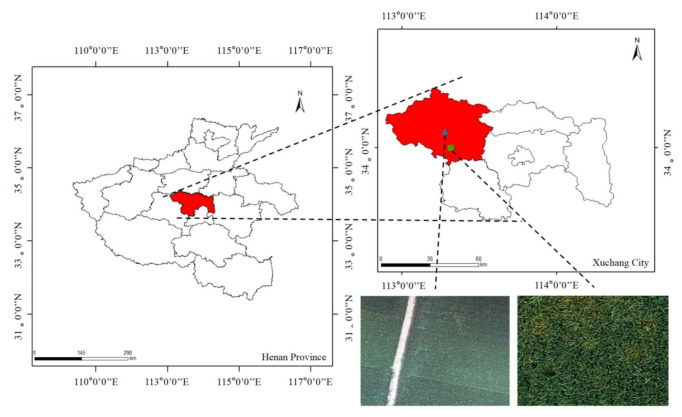
The study area. The left part shows the legend of Henan Province, and the red area indicates Xuchang City. The upper right part shows the legend of Xuchang City; the red area indicates Yuzhou City, the blue triangle indicates the wheat field in Dahuai Village, and the green square indicates the wheat field in Xigolou Village.

In this study, the image data required for the comparison experiments with the SVM, RF, BPNN, FCN, U-Net, and PSPNet algorithms were from a wheat field in Xigolou Village, Yuzhou City, Xuchang City, Henan Province, China (The regional coordinates of wheat stripe rust in this plot are 34°10′23.88″ N, 113°23′31.92″ E), and the image data required for the study of the spatial generalizability of the model were from a wheat field in Dahuai Village (the regional coordinates of wheat stripe rust in this plot are 34°4′56.32″ N, 113°25′23.76″ E); their geographical locations are shown in Figure 1. Yuzhou City is located in the northwestern part of Xuchang City, with a total area of about 1500 km^2^, and more plain areas suitable for winter wheat growth.

We used GPS equipment to investigate the prevalence of wheat stripe rust in Yuzhou City in 2021, recorded detailed information on the growth and geographic location of wheat, and selected the two typical plots mentioned above for research. In addition, the wheat field in Xigolou Village was 9 m^2^ (3 m × 3 m), and the wheat field in Dahuai Village was 2688 m^2^ (42 m × 66 m), totaling about 0.67 acre.

### 2.2. UAV Data and Preprocessing

In this work, a DJI Sentinel 2 UAV (DJI Company, Shenzhen, China) was used to acquire 0.7 cm/px three-channel (RGB) images with the following flight parameters: flight height: 30 m; heading overlap: 90%; side overlap: 90%; and photo mode: isometric. The images were acquired on 12 April 2021, when the wheat was at the nodulation stage, which is the early stage of wheat yellow rust. The acquired image data were postprocessed with PhotoScan v.1.5.1 [24]. Image stitching was used to correct the image sequences with a certain overlap rate in space to form a seamless image with a wide field of view. The five stitching steps included: aligning the photos, building a dense cloud, building a mesh, building a texture, and building an orthomosaic. Due to the limitation of the dialectic relationship between the flight height and spatial resolution of UAVs, a single image is not enough to encompass an entire study area, and stitching of multiple images is usually required to obtain wide-field, accurate aerial images. In addition, stitched images can eliminate the problems of uneven illumination and image deformation caused by different light intensities, inconsistent flight speeds, and differences in flight angles due to wind during the shooting process.

**Figure 2:**
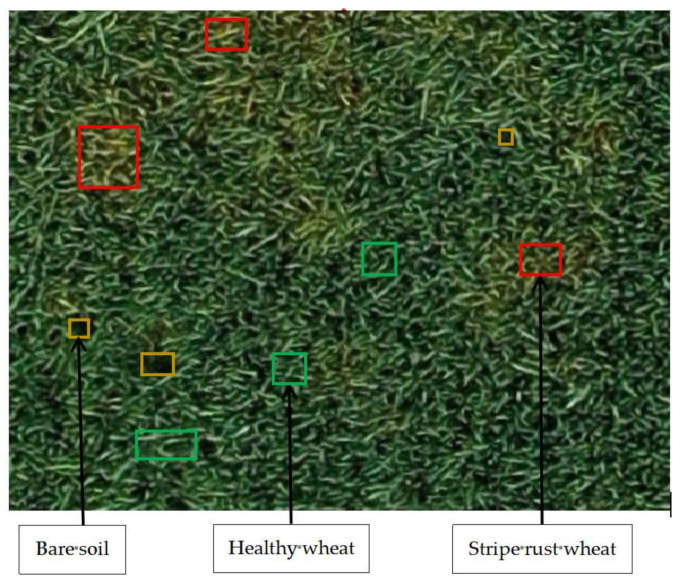
A UAV image with the three categories of samples labeled. The red squares indicate yellow rust wheat, the yellow squares indicate bare soil, and the green squares indicate healthy wheat.

We used an unmanned aerial vehicle (UAV) to collect wheat growth in the study area, mark the details of the different categories, and finally combine the field survey data with the stitched images of the wheat fields in Xigolou Village to complete the sample labeling in ArcMap 10.4.1 [25]. Three categories were marked: healthy wheat, wheat with stripe rust, and bare soil. The annotated category information is shown in Figure 2. We then used the sliding-window method to create the dataset required for model training, with a sliding-window size of 256 × 256 and a step size of 2. In the end, a total of 5580 samples were obtained.

## 3. Method

This study compared the recognition effects of several categories using SVM, RF, BPNN, FCN, U-Net, and PSPNet models on the UAV image dataset acquired from the Xigolou Village plot, investigated the spatial generalization of the PSPNet model with the best recognition effect on the UAV images from the Dahuai Village plot, and finally solved the sample labeling problem based on the weak-sample learning approach. This section focuses on the design scheme of the experiments, the network architecture of the PSPNet semantic segmentation model, the method of weak-sample learning, and the evaluation index of accuracy.

### 3.1. Experimental Design

**Figure 3:**
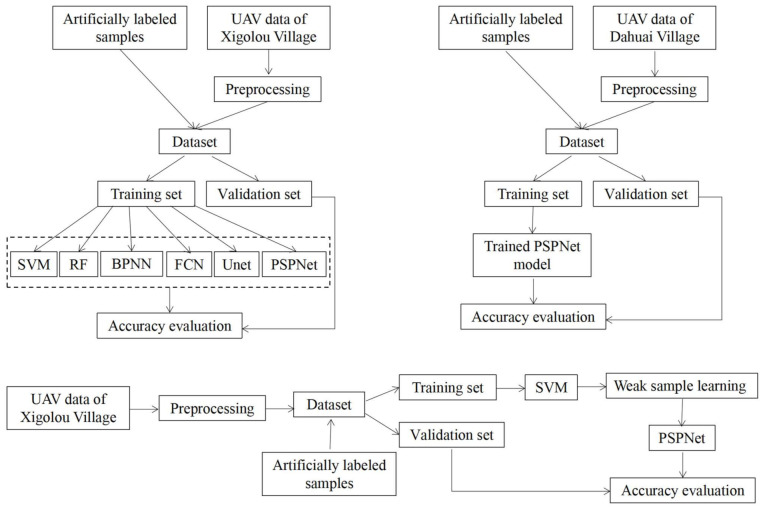
The general roadmap and the experimental components of this study.

Figure 3 presents the general roadmap of this study and describes the experimental components of this study. In order to evaluate the methods of this study, we conducted a series of experiments that were divided into three main parts as follows:

1. Comparison of multiple classification methods (SVM, RF, BPNN, FCN, U-Net, and PSPNet models);
2. Validation of the spatial generalization of neural network models;
3. Studying the feasibility of weak-sample learning methods.

First, we classified the images using SVM, RF, BPNN, FCN, U-Net, and PSPNet models, and compared the classification results in detail to highlight the effectiveness of the semantic segmentation model in identifying healthy wheat, diseased wheat, and bare soil. Then, we verified the spatial generalization ability of the semantic segmentation model with the wheat field images of Dahuai Village in the same period. Finally, based on the problem of label production for large-area images, we proposed to use a weakly supervised learning approach to use the classification results of a higher-precision machine-learning algorithm, SVM, as labels, and then trained and classified them with the PSPNet model, and illustrated the analysis based on the classification results.

### 3.2. Network Architecture

**Figure 4:**
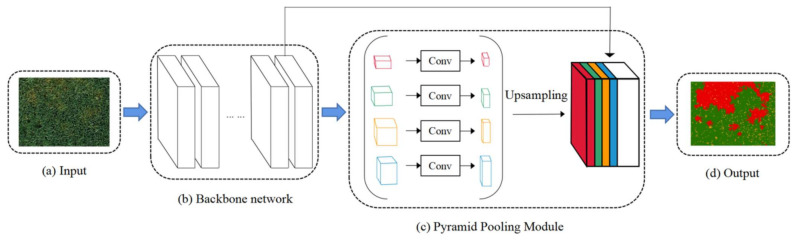
The flow of identification of yellow rust of wheat based on PSPNet: (**a**) a drone image of a wheat field; (**b**) the backbone network of the PSPNet model; (**c**) the pyramid pooling module; (**d**) the predicted output results of the original image, where the red area indicates wheat with yellow rust, the green area indicates healthy wheat, and the yellow area indicates bare soil.

In recent years, with the rapid development of deep learning, the field of crop disease identification has seen a wave of development [26]. Deep convolutional neural networks have been proven to be very effective means and methods. The goodness of image semantic segmentation directly determines the merit of subsequent algorithms for classification or recognition, and scene analysis based on semantic segmentation is considered the most fundamental algorithm [27]. PSPNet is a network architecture that synthesizes multiscale scenes and is divided into two modules: convolutional layer and pyramidal pooling. The workflow of the model is shown in Figure 4. First, the convolutional layer realized the gradual abstraction from the bottom low-level to the high-level features. Second, the pyramid pooling module performed multiscale pooling and convolution of the last layer of abstract features in the convolutional layer module. Then, the multiscale pooled and convolved features were upsampled to maintain the same scale as the last layer of the convolutional layer for joining and convolving the two. Finally, the deconvolution was performed until the scale was consistent with that of the input image, and the attribution of each image element type was determined to achieve the identification of healthy wheat, yellow-rust-afflicted wheat, and bare soil areas.

In this study, we selected an input image size of 256 × 256 with a step size of 2 and an epoch of 50. The backbone model of the PSPNet model was ResNet 34, which contained a jump connection structure that addressed the problem of additional optimization difficulties that could arise with increasing network depth [28]. In addition, the backbone model contained an auxiliary loss function, which could help the model to be better trained. The conv operation in the pyramid pooling module was used to reduce the number of channels by reducing the dimensionality of the convolutional layers of different sizes in order not to underweight the overall feature map. In addition, the upsampling operation in this module was used to align the size with the size of the last layer of the convolutional layer by using bilinear interpolation. The size of the perceptual field could determine to some extent the amount of information about the context that the model could use [29], and the multiscale pooling approach could compensate for the lack of perceptual field, thus increasing the context information in the feature map. This is one of the reasons why the PSPnet model was chosen for this study. In addition, the wheat field contained bare ground areas of arbitrary sizes; these relatively small objects could easily be misclassified, and the neighboring image elements of different classes of objects were not easily identified correctly. Therefore, smaller objects and boundary areas of different classes were paid special attention. Scene analysis provided a complete understanding of the scene and predicted the category of each pixel in the image. The network model’s ability to use both local and global information made the final predictions more reliable.

### 3.3. Weakly Supervised Learning

In recent years, deep learning has been applied to a wide range of industries. In large part, this is due to the availability of deep-learning models that allow practitioners to achieve better results without any manual feature extraction. There is already a large number of state-of-the-art models available, and it can be argued that high-quality models have become a resource that is available to almost everyone. However, there is an easily overlooked problem here; these models rely on a large amount of ground truths. In addition, in many tasks, manual labeling of the training set is both expensive and time consuming. On top of that, tasks often change and evolve in the real world. For example, when the requirements for data labeling change from two categories to four or more categories, the labeling needs to be repeated. Obviously, the workload of this task is huge when relying on manual work alone. Therefore, choosing the method of training deep-learning models by better supervised classification results is a very important task.

Weak-sample learning refers to the use of existing training samples with a small amount of noise to train the model, thus eliminating the labor and time costs of labeling large amounts of data. Regarding the feasibility of using weak samples instead of manually labeled samples, Kaiser et al. [30] affirmed this practice, gave a relevant explanation, and designed a large number of experiments to validate it. In essence, the weak-sample learning approach can be considered as a kind of data augmentation, and there have been numerous studies [31,32,33,34] that showed the robustness of CNN models to sample noise. The high-accuracy classification results from the traditional classification methods are used as labels to train the neural network models, which can yield better classification results, and can achieve efficiency in crop identification or crop disease identification under large areas to some extent.

### 3.4. Accuracy Evaluation

To evaluate the results of wheat yellow rust identification, this study used a confusion matrix for accuracy evaluation. The confusion matrix could clearly show the number of correctly classified features and the number of misclassified categories and features. However, the confusion matrix could not tell the accuracy of each category at a glance, so we needed to derive various classification accuracy indicators from the confusion matrix, including OA, F1 score, and Kappa coefficient, which are the most commonly used indicators. The OA is the ratio of the number of correctly classified category elements to the total number of categories, and the formula is:

(1) $$OA=\frac{\sum_{i=1}^{N}p_{ii}}{P}$$

where *p_ii_* denotes the number of diagonal pixels in the confusion matrix, and *p* denotes the total number of pixels. The Kappa coefficient represents the proportion of error reduction produced by classification versus completely random classification, and the formula is:

(2) $$Kappa=\frac{p\cdot \sum_{i=1}^{N}p_{ii}-\sum_{i=1}^{N}\left(p_{i+}\cdot p_{+i}\right)}{p^{2}-\sum_{i=1}^{N}\left(p_{i+}\cdot p_{+i}\right)}$$

where *p_i+_* and *p*_+*i*_ denote the sum of the pixels in each row and column, respectively, of the confusion matrix. Mean intersection over union (mIoU) is an evaluation index for the performance of a semantic segmentation system. It is used to calculate the ratio of the intersection and union of the true value and the predicted value. The closer the value of mIoU is to 1, the better the effect of the model. The calculation formula for mIoU is as follows:

(3) $$mIoU=\frac{1}{N+1}\sum_{i=0}^{N}\frac{p_{ii}}{\sum_{i=0}^{N}p_{i+}+\sum_{i=0}^{N}p_{+i}-p_{ii}}$$

In addition, the *F*1 *score* can be a better measure of recognition effectiveness and is related to precision and recall. If the precision rate is denoted by *P* and the recall rate is denoted by *R*, the formula for the *F*1 *score* is:

(4) $$F1\;score=\frac{2P\cdot R}{P+R}$$

## 4. Results and Analysis

### 4.1. The Comparison of SVM, RF, BPNN, FCN, U-Net, and PSPNet

**Figure 5:**
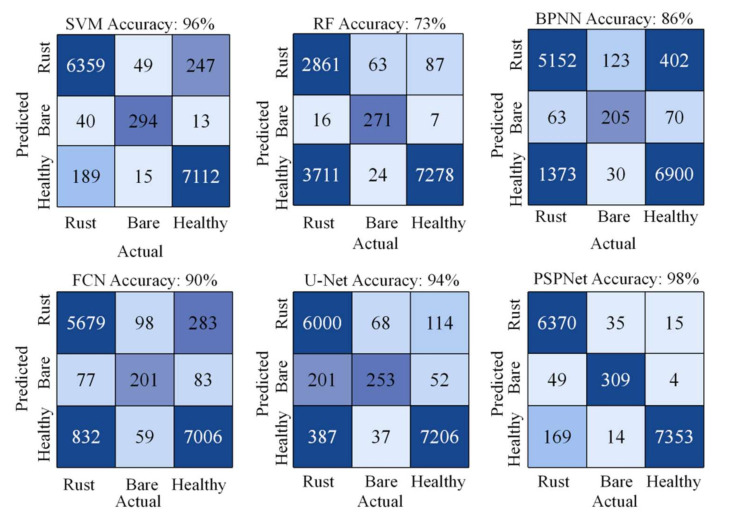
Classification accuracy and confusion matrix of the SVM, RF, BPNN, FCN, U-Net, and PSPNet algorithms.

In this study, the traditional machine-learning classification methods SVM and RF were used to recognize the wheat field images of Xigolou Village, and the classification accuracies were 96% and 73%, the Kappa coefficients were 0.93 and 0.46, and the mIoU values were 86% and 60%, respectively. Obviously, RF was less effective. SVM had the ability to handle large feature spaces and could use inner product kernel functions instead of nonlinear mapping to higher dimensional spaces. RF has been shown to be overfitting on noisy classification problems [35]. In addition, especially for this kind of data with categories that had different values, categories with a larger range of values had a greater impact on the random forest, which did not work well for the task in this study. We selected the structure of BPNN with three hidden layers, the activation function was logistic, and the number of training iterations was 50. The classification accuracy of BPNN was 86%, and the Kappa coefficient was 0.65. The recognition of images under the BPNN algorithm did not achieve high accuracy because the algorithm had fewer network layers and could not fully extract and utilize the features in the images. The mIoU of the FCN model and the U-Net model were 68% and 74%, respectively; the U-Net model incorporated features at different scales and had a skip connection, which ensured that the features recovered from upsampling were more refined [36]. In addition, the classification accuracy of the PSPNet model was 98%, the Kappa coefficient was 96%, and the mIoU value reached 89%. We recorded the classification accuracy and confusion matrix of different algorithms in detail (Figure 5) and summarized the classification results (Table 1). Among these, the value in the confusion matrix represents the number of pixels.

**Figure 6:**
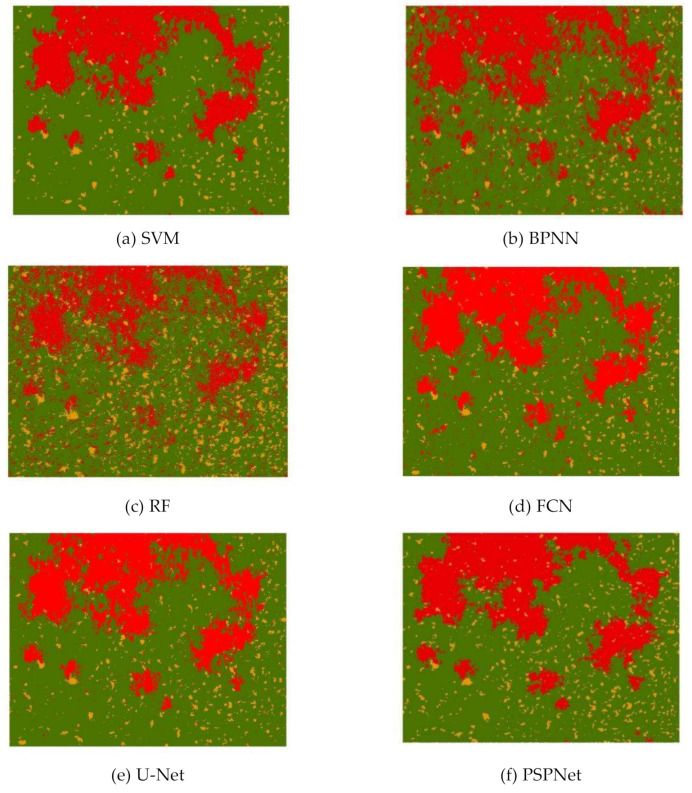
Predicted outputs of the (**a**) SVM, (**b**) BPNN, (**c**) RF, (**d**) FCN, (**e**) U-Net and (**f**) PSPNet algorithms.

Figure 6 shows the predicted output results of the different algorithms. On the whole, the classification result of the SVM algorithm reflected the actual situation of the study area more accurately, except for a slight “salt-and-pepper” phenomenon around the red area. The classification results of the RF algorithm showed a serious salt-and-pepper phenomenon, and the overall effect was very poor. The classification results of BPNN, FCN and U-Net had different degrees of the “salt-and-pepper” phenomenon, and U-Net’s classification results were significantly better than the first two. The PSPNet algorithm’s predicted output map was relatively “clean”, and reflected the actual situation of the study area very accurately.

### 4.2. Spatial Generalization

**Figure 7:**
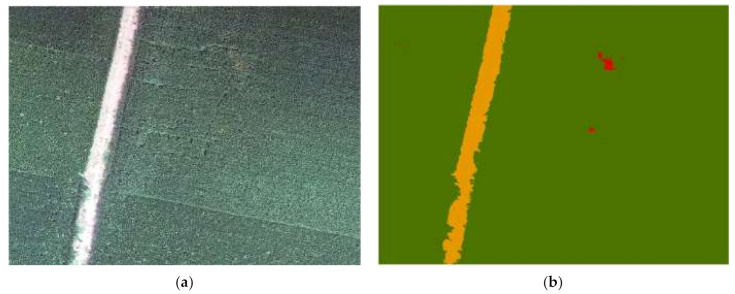
The predicted output of the original image and the PSPNet model. (**a**) is the original figure, and (**b**) is the predicted output result of the model.

Based on the PSPNet semantic segmentation model with good classification results, we used the trained model to perform predictive recognition on another relatively large image that was acquired for a wheat field in Dahuai Village using a UAV on 12 April 2021. The wheat in this period was at the early stage of the development of yellow rust, and in general, less wheat was affected, and the disease was less severe. Figure 7 shows the predicted output of the original image and PSPNet model. It can be seen that there was not an excessive salt-and-pepper phenomenon in the prediction results, and the margins were successful without misclassification. In the original image, it can be seen that the wheat field was densely planted and there was not much bare land; this was reflected in the predicted output image, as the wheat field area on both sides of the road was almost all “green”. The overall classification accuracy of this image was 98%. In summary, it can be seen that the image was classified more accurately. This indicated that the semantic segmentation model selected in this study had some spatial generalization ability.

### 4.3. Weakly Supervised Learning

**Figure 8:**
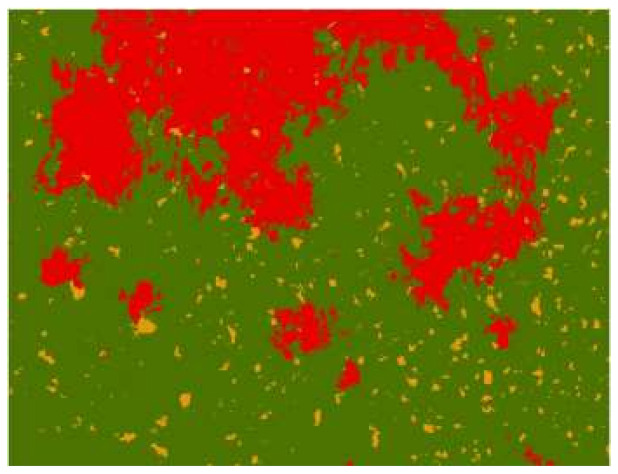
The predicted output of weakly supervised learning.

Based on the traditional classification method of SVM, high-accuracy classification results were obtained with an overall classification accuracy of 96%. Then, we used the classification results as label samples to train the semantic segmentation model and to predict the output of the images obtained from the wheat field in Xigolou Village. A graphic of the overall classification results is shown in Figure 8.

**Figure 9:**
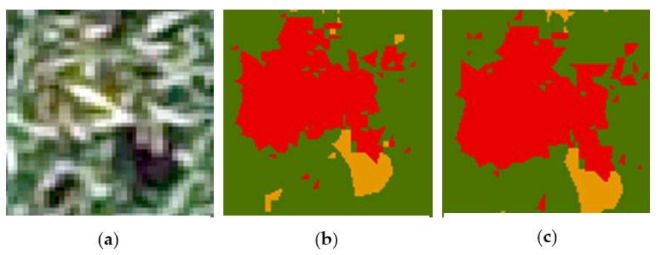
Confusion matrix and classification results for SVM methods and weakly supervised learning methods: (**a**) the original figure; (**b**) the SVM classification results; (**c**) the results generated by the weakly supervised learning method.

Compared with Figure 5, we found that the classification results obtained by weakly supervised learning had slight image blending where the three categories of images were similar. Regarding this point, we selected a typical area containing yellow-rust-affected wheat, healthy wheat, and bare soil, and used the original image of the area, as well as the classification result map of the SVM method and the classification result map of the weak sample supervision method, as shown in Figure 9. It can be seen that the classification result maps produced under the weak-sample supervision method showed misclassification of image elements in the red, yellow, and green areas adjacent to each other. However, the overall recognition effect was still very good. As shown in Table 2, the F1 scores of healthy wheat, wheat with yellow rust, and bare ground were 0.93, 0.80, and 0.95, respectively, and the OA was 94%. This was almost consistent with the accuracy of SVM, which compensated for the shortcomings of the deep-learning model’s high reliance on a great deal of ground truths, and also showed that the weakly supervised learning approach could also obtain high recognition accuracy. This method could maintain good classification performance without using a large number of manually labeled samples.

## 5. Discussion

This section develops relevant discussions based on the presentation and analysis of experimental results. First, the effectiveness of the PSPNet model is discussed in terms of model structure; then, the robustness of the PSPNet model is discussed based on the better recognition results of the model; and finally, the efficiency of the weakly supervised learning method is discussed in the case for which manual labeling of large region samples was not feasible.

### 5.1. The Effectiveness of the PSPNet Semantic Segmentation Model

The PSPNet model contained the jump-connection structure in the backbone model, combined with the hole convolution strategy to extract the feature map, which was overlaid with the fused features with overall information concatenated by the pyramid pooling module, and finally the final pixel-level prediction output image was obtained by the convolution operation. Liu et al. [37] integrated ResNet and a pyramidal scene-resolution network for automated classification of winter wheat from UAV aerial images, and the results showed that the overall recognition accuracy of the model reached more than 90% for winter wheat. Through this study, it can be seen that compared with traditional methods such as SVM and RF, the semantic segmentation model could avoid the shortage of manually subjective selection of effective features, effectively achieve information extraction, and accurately identify healthy wheat, yellow-rust-afflicted wheat, and bare soil. Gao et al. [38] used multilevel semantic features to solve the sparsity problem of roads in remote-sensing images based on the pyramid pooling module of PSPNet. The results showed that the model had a better performance, especially on slender roads. Zhao et al. [39] used the PSPNet algorithm to train samples while considering the different dimensional sizes and coarse texture features of the target objects in large-scale images. The spectral and spatial features of the images were understood by the feature maps extracted in the residual network module. Then, the pyramid pooling module combined the feature information obtained from this module with the feature information obtained from the previous module. Finally, the overall classification accuracy of the satellite images of land covering the Beijing–Tianjin region of China was over 80%. In general, the PSPNet semantic segmentation model is an end-to-end CNN model that takes an image as input and outputs its classification results. The model is able to learn texture information in the image, which is the key information for identifying different categories in the image. In this study, the classification results of the SVM and RF algorithms showed different degrees of a “pretzel” phenomenon, among which the latter showed more serious effects. The BPNN algorithm had a simpler structure, and most of the misclassification results were in the vicinity of different classes of image elements, while the PSPNet algorithm had a cleaner classification result with the highest overall accuracy of 98%. Schirrmann, Michael et al. [40] used the ResNet model to identify wheat stripe rust images taken by RGB cameras with a total accuracy of 90%. Hayit, Tolga et al. [41] artificially planted wheat and inoculated with Urediniospore suspension, then used a camera to photograph wheat leaves to create a dataset, and identified them based on the Xception model. The overall accuracy rate reached 91%. Compared with these, the recognition accuracy achieved by the PSPNet model was relatively high. Deep learning is more sensitive to the shape characteristics of the sample. After wheat is infected with stripe rust, it will not only cause yellowing of the leaves, but also the distribution of spores on the leaves, so the characteristics are more obvious. In addition, we obtained drone images of a naturally mature wheat field (32°29′48.12″ N, 115°10′28.67″ E) in Huaibin County, Xinyang City, Henan Province on 30 May 2021. The area is 900 square meters (30 m × 30 m), and at that time, the wheat had chlorosis. Through additional experiments, it was found that this study could better distinguish wheat chlorosis caused by stripe rust from wheat chlorosis caused by normal maturity, with an accuracy of 98% and an mIoU of 83%. In the follow-up, we will investigate more types of wheat diseases and images with richer bands for related research.

### 5.2. Spatial Generalization Capability of the Model

Spatial generalizability means training a model using images and category labels from a certain place and period, and later applying the trained model to extract that category from other locations for which no training data is available. Therefore, the essence of generalization is to construct models that can extract features from a limited training set, so that the feature representation has good flexibility and stability. In practical production, it is often difficult to collect a large range of sample labels, and using a model with high generalization capability can substantially reduce the time and human resources consumed by this process. Wang et al. [42] proposed that the training samples collected in reality could not cover various situations, so the algorithm with good generalization performance is more adaptable to multiple situations in practice. Gao et al. [43] improved the traditional algorithm based on convolutional neural network as a way to classify and recognize SAR images, and improved the generalization of the model. Liang et al. [44] proposed a recognition model with a multilevel convolutional feature pyramid to solve the problem of poor generalization in the recognition and classification of food images. Yang et al. [45] used convolutional neural networks for training, and demonstrated the stability and generalization of the method by analyzing the training results of a synthetic dataset and a dataset of real soybean seeds. In this study, the semantic segmentation model PSPNet was trained using the UAV images and corresponding category labels of Xigolou Village in Yuzhou City, Henan Province in 2021 as the training set. The trained deep-learning model was used to predict the output of the UAV image data from the same time but different plots, and the overall accuracy of its prediction results reached 98%, which proved that the semantic segmentation model had certain spatial generalizability.

### 5.3. The Efficiency of Weakly Supervised Learning

In recent years, semantic segmentation models have made progress in various fields such as medical and road extraction. Semantic segmentation algorithms are dedicated to identifying the category of each pixel in an image, and this method requires corresponding pixel-level annotations for training. A large number of labeled samples is a prerequisite for deep learning, which is precisely the current bottleneck of deep learning for remote-sensing big data. At present, in remote-sensing recognition of crop diseases, almost all of the methods rely on manually labeled samples, and no other methods have been adopted. In this study, we proposed to solve this problem by using weak-sample learning. In weakly supervised learning algorithms, pixel-level recognition and classification of images can be performed using weak labels, and such data inevitably contain some errors, but it had been shown that weak-sample supervised algorithms were quite robust to noise in weak labels, which also laid the foundation for avoiding labeling large amounts of data [30]. Ma et al. [46] used the scene-level data labels as the supervised information of training samples for road extraction of remote-sensing images based on a weakly supervised deep convolutional network method. In this study, the high-precision recognition result (96%) of the traditional classification method SVM for the wheat field in Xigolou Village was used as a weak sample to train the PSPNet semantic segmentation model, and a prediction result of 94% was obtained, which solved the problem of time-consuming and labor-intensive labeling a large number of samples to some extent. At the same time, the results also showed that the crop classification results obtained based on the traditional classification algorithm as labeled samples could also be used to train the deep-learning model with high recognition accuracy, which proved the feasibility of using the traditional classification results as weak samples.

## 6. Conclusions

In this study, it was proposed to identify wheat stripe rust based on UAV images using the PSPNet semantic segmentation model, and the spatial generalization of the model was further verified. In addition, for the sample annotation problem, a weak-sample supervision approach was proposed to better solve this problem. To demonstrate the efficiency of the PSPNet model, we compared it with SVM, RF, BPNN, FCN, and U-Net algorithms, and the results showed that the PSPNet model had the highest overall accuracy, the largest Kappa coefficient, and the best recognition effect. Further, the generalizability of the model was investigated using another wheat field image from the same period. Since the UAV images used in this study were acquired in April 2021, when wheat was in the plucking stage and the symptoms of stripe rust in wheat were light, there were not an excess of red areas in the classification result map, and most of them were green areas. In the spatial generalization study, we obtained a more accurate classification result map and a higher overall classification accuracy. Considering various problems, including the time-consuming, laborious, and large human errors associated with manual labeling of sample data, we proposed an efficient solution to this problem in the crop disease identification task using weak-sample supervision. In this study, the SVM algorithm obtained high classification accuracy, and we used the classification results of this algorithm directly as labels for training the deep-learning model; although this had some errors, it did not pose a large threat to the overall task, and the final classification results showed that the weak-sample supervision method had some reliability and efficiency.

Overall, this study provided a new idea for using deep learning to achieve large-scale crop disease identification. Future work will be to validate the transferability of this study and the feasibility of the weak-sample supervised approach in more UAV RGB image data with different crops and different diseases. In addition, we will also further introduce hyperspectral UAV images for deeper studies on crop diseases.

## Figures and Tables

**Table 1:** Classification performance of the SVM, RF, BPNN, FCN, U-Net, and PSPNet algorithms.

Method	Accuracy	Kappa	mIoU
SVM	96%	0.93	86%
RF	73%	0.46	60%
BPNN	86%	0.65	64%
FCN	90%	0.81	68%
U-Net	94%	0.89	74%
PSPNet	98%	0.96	89%

**Table 2:** The results for weakly supervised learning.

Classification Result	Ground Truth		
	Rust Wheat	Bare Area	Health Wheat
Rust wheat	6043	58	271
Bare area	37	274	18
Healthy wheat	508	26	7083
F1 score	0.93	0.80	0.95
OA = 94%			

## Data Availability

Not applicable.
